# A case of nocardiosis in a patient with ulcerative colitis on chronic corticosteroids, infliximab, and upadacitinib

**DOI:** 10.1002/ccr3.7362

**Published:** 2023-05-16

**Authors:** Benjamin M. Moy, Abhishek Shenoy, Leslie B. Aldrich

**Affiliations:** ^1^ University of Michigan Medical School Ann Arbor Michigan USA; ^2^ Division of Gastroenterology and Hepatology, Department of Internal Medicine Michigan Medicine Ann Arbor Michigan USA

**Keywords:** Crohns, IBD, immunosuppression, JAK inhibitors, Nocardia

## Abstract

**Key Clinical Message:**

Immunosuppression, malnutrition, and underlying infection can unmask obscure infections which can be challenging to identify. Early diagnosis and treatment of infections in immunosuppressed patients are essential due to high morbidity and mortality.

**Abstract:**

The immunosuppressive effects of treatment for ulcerative colitis (UC), including chronic corticosteroids, anti‐TNF agents, and JAK inhibitors, can impact the spread of latent or obscure infections. Clinicians should have a low threshold for pursuing aggressive diagnostic and therapeutic intervention in patients who show signs of clinical deterioration while on immunosuppressing medications. Our unique case highlights an immunosuppressed patient with UC who developed Nocardiosis after initiation of upadacitinib while hospitalized for concurrent UC flare and *Clostridium difficile* infection.

## Graphical abstract

We report a complex infection of Nocardiosis and C. difficile colitis that developed in a patient with ulcerative colitis in the setting of chronic immunosuppression and malnutrition.

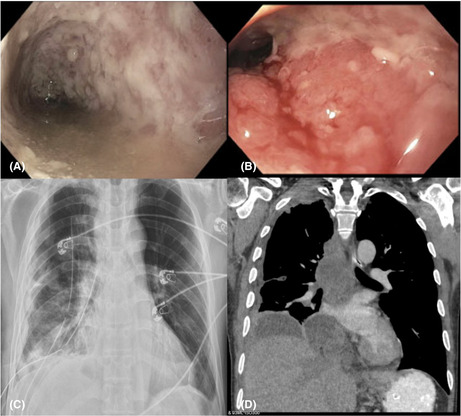

## 1 INTRODUCTION

Small molecule Janus kinase (JAK) inhibitors such as tofacitinib (Xeljanz^Ⓡ^) and upadacitinib (Rinvoq^Ⓡ^) have emerged as efficacious treatments for ulcerative colitis (UC) but also suppress immunologic responses in patients by downregulating inflammatory pathways. Nocardiosis is a disease caused by Gram‐positive, partially acid‐fast, methenamine silver‐positive, aerobic actinomycetes. It causes pulmonary, skin, or disseminated manifestations in immunosuppressed patients, with the most common organisms being *Nocardia asteroides and Nocardia brasiliensis*.

Nocardiosis has been classically diagnosed in patients with prior HIV infection, organ transplantation, or long‐term corticosteroid use. We report a rare case of nocardiosis in a patient with UC initiated on upadacitinib for severe UC on a home regimen of chronic corticosteroids and an anti‐TNF agent. We describe the diagnostic and therapeutic workup for severe infection secondary to immunosuppression.

## 2 CASE PRESENTATION

**FIGURE 1:**
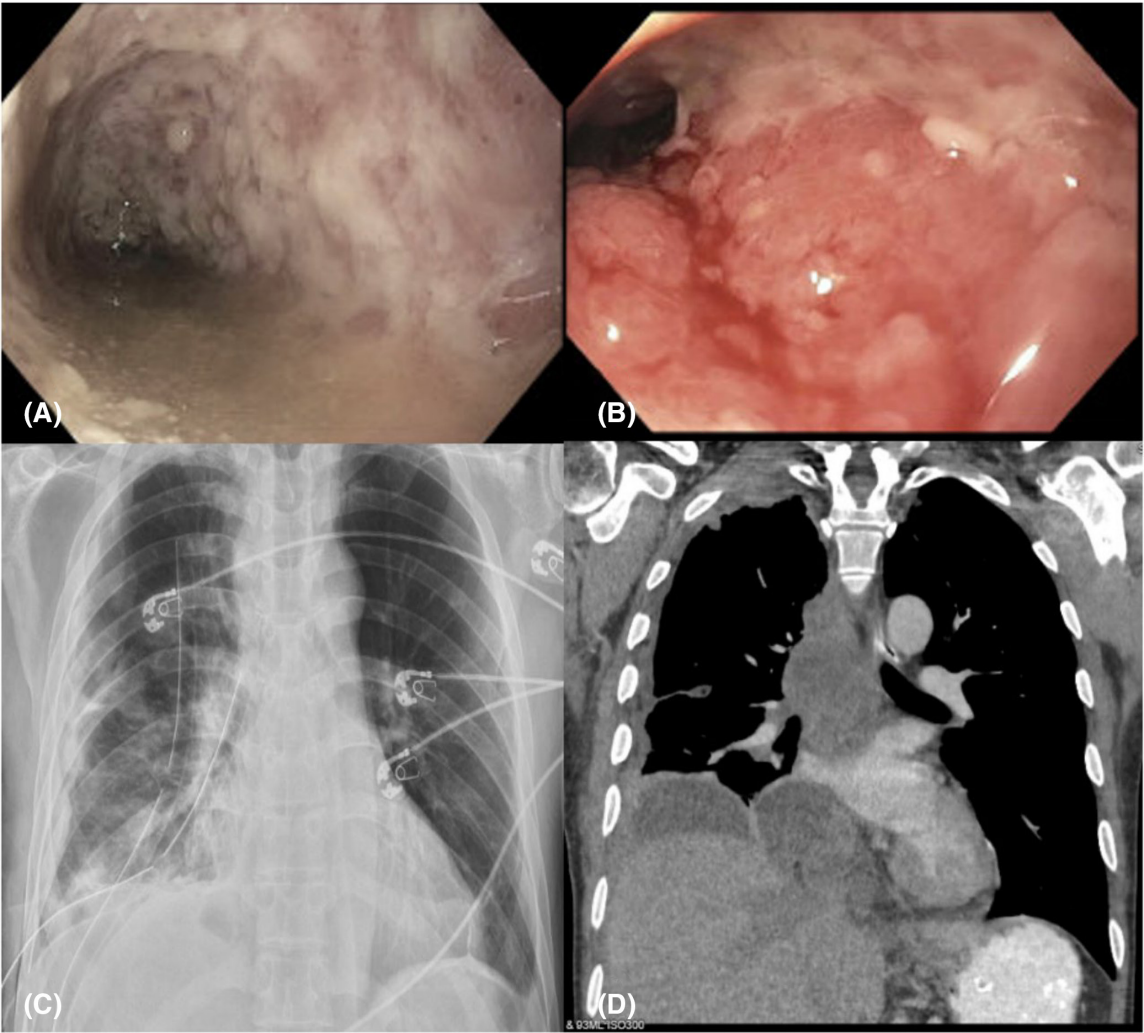
Imaging shows pseudomembranes suggesting *Clostridium difficile* colitis and empyema with cultured *Nocardia*. (A) Flexible sigmoidoscopy shows pseudomembranes in the sigmoid colon consistent with *C*. *difficile* colitis. (B) Flexible sigmoidoscopy shows ulcerations and pseudomembranes in the sigmoid colon. (C, D) Chest X‐ray and CT show worsening large right‐sided pleural effusion 48 h after initiation of upadacitinib.

A 47‐year‐old male patient with UC on a home regimen of 10 mg prednisone and 100 mg infliximab‐abda (Renflexis^Ⓡ^) presented with >30 dark, loose stools daily, confusion, weight loss of five pounds in 2 weeks, chills, and dyspnea. He also reported right flank pain for over a week and a cough lasting several months. His social history was relevant for extensive exposure to soil, mulch, and wood chips from his occupation. He also had a prior 11.5‐pack‐year smoking history. His physical exam at the time of admission was remarkable for fatigued appearance and crackles to auscultation of the right lung. Laboratory values at the time of admission were remarkable for a white blood cell count of 8.0 WBCs/μL, hemoglobin 11.2 g/dL, sodium 125 mEq/L, erythrocyte sedimentation rate (ESR) >130 mm/h (reference range: 0–15 mm/h), C‐reactive protein (CRP) of 36.4 mg/dL (reference range: 0.0–0.6 mg/dL), positive *Clostridium difficile* toxin A/B and enzyme immunoassay, and negative urinalysis. Flexible sigmoidoscopy showed friability and contact bleeding favoring UC flare (Mayo Score 3) and the presence of pseudomembranes suggesting concurrent *C. difficile* pancolitis (Figure 1A, B). The presence of chronic active colitis consistent with UC was confirmed on pathology. A chest X‐ray and CT chest revealed a large, loculated, right‐sided pleural effusion, a chest tube was placed, and he received vancomycin and piperacillin‐tazobactam. Given that his initial pleural effusion cultures remained negative and his right‐sided pleural effusion had decreased in size, the chest tube was removed after 5 days. His antibiotic regimen was transitioned from piperacillin‐tazobactam to cefazolin, and he was started on 30 mg methylprednisolone and 30 mg upadacitinib twice daily for UC.

Though our patient improved in the initial 24–36 h of upadacitinib therapy, 2 days after initiation, he developed a fever of 102 F and tachycardia. A chest CT revealed a worsening large multiloculated right‐sided pleural effusion with posterolateral, paramediastinal, and subpulmonic components, scattered irregular nodular opacities, and right hilar and mediastinal lymphadenopathy (Figure 1C,D). Upadacitinib was held and he underwent right‐sided thoracoscopic decortication with the placement of two chest tubes. Cultures from this effusion grew *Nocardia farcinica* and *Nocardia kroppenstedtii* although the acid‐fast bacilli (AFB) smear was negative. He was started on linezolid and trimethoprim‐sulfamethoxazole and continued vancomycin for *C. difficile* colitis. At the recommendation of the infectious diseases service, an MR Brain ruled out intracranial spread, as *Nocardia* commonly disseminates to the brain. After a repeat CT chest/abdomen/pelvis showed improvement in his empyema, one chest tube was removed and the other was replaced with an empyema drain.

He was discharged on trimethoprim‐sulfamethoxazole and linezolid for the *Nocardia* empyema, vancomycin for *C. difficile* colitis, and a prednisone taper for UC. He transitioned from infliximab‐abda to vedolizumab (Entyvio^Ⓡ^) upon discharge for UC maintenance therapy. Ultimately, the patient's UC symptoms improved significantly on vedolizumab, and his empyema and *C. difficile* colitis gradually resolved on long‐term antibiotic therapy.

## 3 DISCUSSION

UC medications, including chronic corticosteroids and agents modulating tumor necrosis factor (TNF), interleukins (IL), or Janus kinases (JAK), are known to increase the risk of infection due to their immunosuppressive effects. Upadacitinib is an effective medication targeting JAK‐1 in patients with UC. In phase 2b trials of upadacitinib, serious infections occurred in 2% and 3.6% of patients on a daily dose of 15 and 45 mg upadacitinib, respectively.^1^ Patients in these trials were permitted to be treated concomitantly with oral aminosalicylates, methotrexate, and oral corticosteroids at stable doses, and patients treated concomitantly with biologics, intravenous corticosteroids, and other immunosuppressants were excluded.^1^ Although the rates of serious infection on upadacitinib are relatively low, our patient was already immunosuppressed while on his home prednisone and infliximab‐abda and thus, may have had a higher propensity to developing a serious infection than described in the aforementioned rates.

*Nocardia* are Gram‐positive, partially acid‐fast, methenamine silver‐positive, aerobic bacteria that primarily affect the lungs, brain, and skin.^2^ Although the patient's AFB smear was negative, *Nocardia*, as well as additional bacteria like *Mycobacterium*, *Corynebacterium*, *Rhodococcus*, *Gordona*, and *Tsukamurella*, can all be variably acid‐fast on appropriate staining. Because *Nocardia* is primarily soil‐borne, our patient was likely exposed to *Nocardia* given his work in agriculture. Co‐infection with *Nocardia* with other pathogens, including SARS‐CoV‐2, cytomegalovirus (CMV), HIV, *Aspergillus, Pneumocystis jirovecii* (PJP), and *Mycobacterium avium* complex (MAC), has also been reported.^2^, ^3^, ^4^, ^5^ Several cases of nocardiosis have been specifically reported in patients taking medications for IBD, including corticosteroids, biologics like infliximab and golimumab, and immunomodulators like tofacitinib.^2^, ^5^, ^6^, ^7^, ^8^ However, these cases are isolated reports, and the true likelihood of nocardiosis is not well characterized in this patient group.

The patient's existing immunosuppression of infliximab‐abda and chronic corticosteroid regimen may have contributed to the existing effusion that developed before upadacitinib was started. Generally, drainage of an effusion to the point that the chest tube can be removed is the standard of care for resuming anti‐TNF therapy. Thus, upadacitinib was only started after ensuring that the patient did not develop new fevers, blood cultures remained negative, the effusion size was decreasing based on chest radiographs, and the chest tube was successfully removed. It is unlikely that the patient's effusion worsened solely due to the incorporation of upadacitinib for UC treatment. A more plausible explanation may be that a combination of factors, including prior chronic corticosteroid use, malnutrition, inadequate chest tube drainage, and treatment with infliximab, all contributed to a clinical picture that influenced the spread of *Nocardia*.

When an abscess or empyema is drained with a chest tube, it is critical to ensure adequate drainage before initiating or resuming UC treatment. Although it is common to start anti‐TNF agents when adequate drainage is achieved, such a strategy may be more complex for starting JAK inhibitors, especially when the patient is already immunosuppressed. Initial clinical improvement should be interpreted cautiously and does not necessarily indicate readiness to introduce or resume immunosuppressive agents.

The incidence of *Nocardia* infection is low; nevertheless, early diagnosis and treatment in immunosuppressed patients are essential due to its high morbidity and mortality. Latent or obscure infections as in the case described are challenging to identify in advance. Clinicians should have a low threshold for pursuing aggressive diagnostic and therapeutic intervention in patients who show signs of clinical deterioration while on chronic corticosteroids or UC treatments such as anti‐TNF agents or JAK inhibitors. Clinicians should also consider a wide range of infectious agents in these scenarios.

## AUTHOR CONTRIBUTIONS

**Benjamin M. Moy:** Conceptualization; data curation; formal analysis; investigation; project administration; supervision; writing – original draft; writing – review and editing. **Abhishek Shenoy:** Conceptualization; data curation; formal analysis; supervision; writing – review and editing. **Leslie B. Aldrich:** Conceptualization; writing – review and editing.

## FUNDING INFORMATION

None.

## CONFLICT OF INTEREST STATEMENT

The authors do not report any conflicts of interest.

## CONSENT

Written informed consent was obtained from the patient to publish this report in accordance with the journal's patient consent policy.

## Data Availability

Data sharing is not applicable to this article as no new data were created or analyzed in this study.
